# Personalized medicine in human space flight: using Omics based analyses to develop individualized countermeasures that enhance astronaut safety and performance

**DOI:** 10.1007/s11306-013-0556-3

**Published:** 2013-06-27

**Authors:** Michael A. Schmidt, Thomas J. Goodwin

**Affiliations:** 1 Advanced Pattern Analysis & Countermeasures Group, MetaboLogics. LLC, Infectious Disease Research Complex, Colorado State University, 3185 Rampart Road, Fort Collins, CO 80521 USA; 2 NASA Johnson Space Center, Disease Modeling and Tissue Analogues Laboratory, Biomedical Research and Environmental Sciences Division, Houston, TX 77058 USA

**Keywords:** Omics, Genomics, Proteomics, Transcriptomics, Metabolomics, Personalized medicine, Space flight, Human, Astronaut health, Exploration, Systems biology, Single nucleotide polymorphism, Oxidative stress, Human performance, Essential inputs, Micronutrient, DNA stability, DNA repair

## Abstract

Space flight is one of the most extreme conditions encountered by humans. Advances in Omics methodologies (genomics, transcriptomics, proteomics, and metabolomics) have revealed that unique differences exist between individuals. These differences can be amplified in extreme conditions, such as space flight. A better understanding of individual differences may allow us to develop personalized countermeasure packages that optimize the safety and performance of each astronaut. In this review, we explore the role of “Omics” in advancing our ability to: (1) more thoroughly describe the biological response of humans in space; (2) describe molecular attributes of individual astronauts that alter the risk profile prior to entering the space environment; (3) deploy Omics techniques in the development of personalized countermeasures; and (4) develop a comprehensive Omics-based assessment and countermeasure platform that will guide human space flight in the future. In this review, we advance the concept of personalized medicine in human space flight, with the goal of enhancing astronaut safety and performance. Because the field is vast, we explore selected examples where biochemical individuality might significantly impact countermeasure development. These include gene and small molecule variants associated with: (1) metabolism of therapeutic drugs used in space; (2) one carbon metabolism and DNA stability; (3) iron metabolism, oxidative stress and damage, and DNA stability; and (4) essential input (Mg and Zn) effects on DNA repair. From these examples, we advance the case that widespread Omics profiling should serve as the foundation for aerospace medicine and research, explore methodological considerations to advance the field, and suggest why personalized medicine may become the standard of care for humans in space.

## Introduction

To date, more than 600 civilians have registered to fly into space aboard commercial suborbital space vehicles (D. Durda, personal communication, June 3, 2013). That is more than the 534 individuals that have been in Earth orbit, since space flight began in the 1960s (NASA, NP-2005-01-001 JSC, NASA 2005, List of Space Travelers by Name 2013). Currently, there are ambitious private sector plans to send humans to space for mining and other high risk commercial endeavors.

With increasing space flight participation comes increasing heterogeneity in the flying population, extended duration missions, and prolonged exposure to the high radiation environment beyond low Earth orbit (BLEO). Highly variable risk profiles, coupled with the unusually hostile conditions of space will force us to adopt new human factors approaches that are commensurate with these demands.

For instance, recent evidence has shown that some 20–25 % of the long duration astronauts on the International Space Station (ISS) developed persistent ocular problems after missions of varying lengths, especially those missions >4 months (Smith et al. 2012; Zwart et al. 2012). With this outcome, one is compelled to consider that unique susceptibilities may exist in the affected group and that foreknowledge of such susceptibility allows for the development of protective or adaptive countermeasures. Indeed, there is preliminary evidence this astronaut sub-population may have altered one carbon metabolism, with specific genetic and micronutrient attributes, the effects of which are further exacerbated by the space flight environment.

**Fig. 1:**
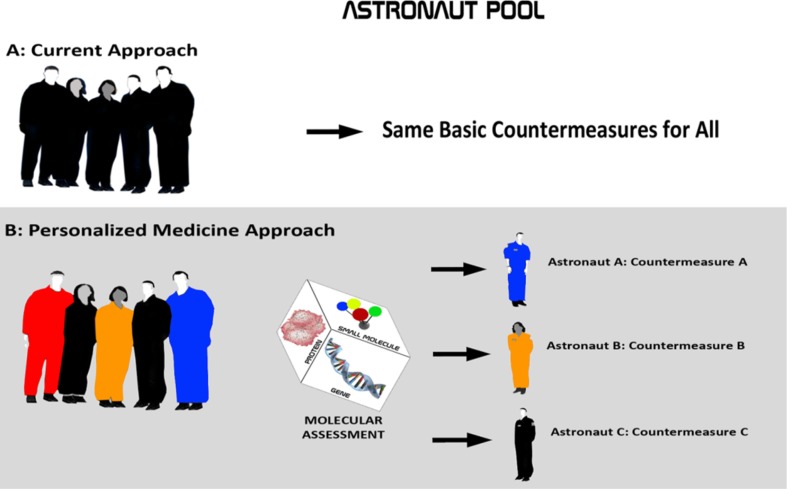
Personalized medicine paradigm for humans in space. **a** Astronauts today receive advanced medical care, but they do not undergo comprehensive genotyping, metabolic network assessment, and assessment of essential small molecule inputs for the purpose of developing countermeasures. Countermeasures are generalized across the astronaut pool. **b** Under the proposed new paradigm, a comprehensive assessment of targeted genome, transcriptome, proteome, and metabolome will guide countermeasure development, with each astronaut receiving a countermeasure package tailored to the individual and to the mission

In order to preserve and enhance performance and safety in the evolving astronaut, space scientist, and space tourist populations, space biomedicine will increasingly be forced to understand individual risk profiles and to develop countermeasures that are tailored to each individual participant (Fig. 1).

This necessarily drives us toward the emerging field of personalized medicine. Personalized medicine uses information about a person’s genes, proteins, and metabolites in the context of his or her diet, nutrition, lifestyle, and environment to prevent, diagnose, and treat or prevent disease. In relation to space flight, one can further state that personalized medicine uses information about a person’s genes, transcripts, proteins, and metabolites in the context of his or her diet, nutrition, lifestyle, and environment to optimize safety and performance commensurate with the demands of a unique condition, habitat, or mission profile.

Today and in the future, personalized medicine approaches will afford us the ability to individualize countermeasures in an effort to improve astronaut performance and safety. This is not aimed at restricting mission participation. On the contrary, the approach is focused on understanding individual astronaut profiles and on developing countermeasures that optimize the astronaut’s ability to participate at his or her highest functional level.

In the field of human space flight, personalized medicine has become possible, as advances in genomics, transcriptomics, proteomics, metabolomics, and bioinformatics affords us the ability to characterize individual profiles with low cost and high precision. Because of the small number of individuals, the high risk of mission failure, and the extreme demands upon individuals, one can argue that human space flight is among the most obvious environments in which to deploy personalized medicine.

This review will summarize: (1) key elements of personalized medicine that are germane to human space flight, (2) knowledge that will allow us to deploy components of personalized medicine today, and (3) recommendations for evolving a sophisticated platform to advance personalized medicine for the future of human space flight.

Our goal is to address some of the most challenging issues facing humans in space, and to explore how individual differences may increase or decrease risk under these unique conditions.

## Background: systems biology, functional genomics, gene variants, and drug metabolism in space biomedicine

### Applied systems biology in space biomedicine

Systems biology can be described as the study of the interactions between the components of *biological systems*, and how these interactions give rise to the function and behavior of that system. In human medicine, systems biology traditionally incorporates genomics, transcriptomics, proteomics, and metabolomics. Data derived from these Omics disciplines are combined with metadata about the subject’s: (1) environment, (2) diet, (3) nutrition, (4) psychosocial dynamics, (5) lifestyle, (6) medical treatments, (7) antecedents, (8) anthropometrics, and, in general, (9) broad information about phenotype. Multivariate analysis and modeling methods are applied to the high dimensional data sets derived from these analyses, in order to describe systems, derive meaning, and generate new hypotheses.

In the current space biomedicine paradigm, targeted, hypothesis-driven methods are the rule. In most cases, molecular targets to be analyzed are preselected, based on a guiding hypothesis. This method has served space biomedicine well for the duration of its history. However, it is notably limited by the fact that targeted-analyses, by design, fail to detect off-target, or unanticipated effects. The strength of Omics-based, data-driven approaches is that they afford us the ability to detect unanticipated effects, reveal novel pathways and mechanisms, and generate new hypothesis, from which follow up targeted analyses can be performed. This can shorten the discovery time from decades to years and allow us much more rapid development of countermeasures for these extreme conditions. This is particularly relevant to human space flight, because there are so few subjects.

The extreme condition of space and the complexity of human systems warrant an emergent approach that is rooted in the new systems biology paradigm. Under this emergent model, test conditions of astronauts in Earth-based analogue studies and actual mission conditions would be studied using non-targeted and targeted Omics-based methods. These methods can be used to develop assessment and countermeasure protocols specific to each astronaut, which would form the basis of personalized medicine.

In this review, we build a case for Omics-based medicine in human space flight that is personalized, preventive, and predictive. Rather than being a comprehensive overview of the field, our discussion is centered on five concepts that represent components of the systems biology paradigm, which have immediate and near-term application in space biomedicine. These include: (a) genetic variants that may drive unique risk profiles of astronauts in the general space environment; (b) genetic variants that may alter risk and efficacy profiles of therapeutic drugs deployed in space; (c) essential inputs into metabolism that may raise or lower individual risk profiles in space; (d) molecular variants that may alter individual risk profiles in the high radiation environment of space; and (e) how combinatorial events may aggregate to shift risk profiles.

### Functional genomics and space biomedicine

The emergence of personalized medicine has been inspired, in part, by compelling advances in genomics applied to diagnostics and therapeutics in oncology. For instance, molecular diagnostic tests for human epidermal growth factor receptor 2 (HER2) are used to identify the patients who will benefit from receiving trastuzumab (Herceptin^®^, Herclon^®^) and other drugs that target HER2, such as lapatinib (Tykerb^®^). Over-expression of HER2 has been shown to play an important role in the progression and pathogenesis of certain aggressive types of breast cancer. More recently, it has emerged to become an important biomarker that aids in therapeutic guidance (Tanaka 2010).

Two complex diagnostic tests, Onco*type* DX^®^ and MammaPrint^®^, use genetic information to help physicians chart the best course of treatment for breast cancer patients. Onco*type* DX^®^ can determine whether women with certain types of breast cancer are likely to benefit from chemotherapy (Tanaka 2010). MammaPrint^®^ can determine which early-stage breast cancer patients are at risk of distant recurrence following surgery (Pers Med Coalition 2010).

Both tests place patients into risk categories that inform physicians and patients whether their cancer may be treated successfully with hormone therapy alone or whether a more aggressive treatment is needed.

Current advances in systems biology and personalized medicine now afford us the ability to apply similar sophistication to astronauts entering the extreme conditions of space. Below, we characterize a *subset* of genomic and small molecule variants that are highly relevant to human space flight and human performance. We have limited our discussion to these in order to simplify the discussion for the purpose of this review. Beyond these examples, there is extensive literature on an array of molecular variants that are relevant to space flight today, in addition to a growing list that we envision will become relevant with further elucidation.

For the purposes of this review, we will explore, by example (1) gene and small molecule variants associated with metabolism of therapeutic drugs used in space; (2) gene and small molecule variants associated with one carbon metabolism; (3) gene and small molecule variants associated with iron metabolism, oxidative stress, and DNA stability; and (4) elemental essential input that bears upon energy regulation, DNA repair, and oxygen utilization in space. We conclude by examining a path whereby systems biology may become the foundation for developing personalized medicine in human space flight.

The purpose this review is not to offer a policy document, but rather to explore a series of core concepts from which personalized approaches to enhance astronaut performance, endurance, and safety can be developed.

## Gene variants and drug metabolism in space

During missions, astronauts are provided with an array of prescription drugs that are deployed based on Earth-based clinical need. In the past, little consideration has been given to the potential for adverse drug reactions, based on individual genotype. However, we must now consider two important aspects of personalized drug responses in astronauts on prolonged missions. These include the potential for adverse drug reactions and the potential that drug efficacy can be negatively impacted by the biochemical uniqueness of the astronaut and his environment. Either or both can contribute to adverse health outcomes and altered astronaut performance in space. Because of the substantial variance possible in individual responses, it becomes imperative that we characterize the variants in astronauts in order to limit adverse effects on exploration missions.

While some prescription drug use will be pre-planned based on astronaut clinical needs, the deployment of pharmaceuticals in space flight will often be based on circumstantial need. Aerospace medicine physicians will want to ensure that the risk of a pharmaceutical triggering an adverse drug reaction in space is kept to a minimum, as extensive medical facilities will be unavailable. This can be done by understanding the specific drug metabolism profiles of each individual and identifying by genotypic assessment the drugs most likely to be poorly tolerated in each astronaut. This profile can then be used to design the optimum personalized drug profile, should pharmaceutical intervention be required.

The optimum drug profile can be developed by characterization of Phase I (cytochrome P450) and Phase II drug metabolism profiles.

### Personalized drug regimens based on cytochrome P450

The first such characterization of individual astronaut biotransformation capacity should be based on individual cytochrome P450 (CYP450) profiles. The cytochrome P450 family (CYP450) is a major subset of drug-metabolizing enzymes. The CYP450 family of enzymes includes, but is not limited to, the following important genes (Wu 2011).

- CYP 2D6 is also known as debrisoquine hydroxylase, which catalyzes the oxidation of approximately a quarter of all the commonly used therapeutic drugs in used clinical practice today. For instance, codeine is metabolized by CYP2D6 to morphine. In such cases, enhanced CYP2D6 activity (i.e., in CYP2D6 ultra-rapid metabolizers) predisposes one to opioid intoxication (Gasche et al. 2004).
- CYP 2C19 (*S*-mephenytoin hydroxylase) acts on weakly or strongly basic drugs containing one hydrogen bond donor, or if there are functional groups containing carbon or sulfur double bonded to oxygen present in the substrate. CYP 2C19 is responsible for the metabolism of anticonvulsant drugs, proton pump inhibitors, and drugs that inhibit platelet function.
- CYP3A4 involved in the oxidation of the largest range of substrates of all the CYPs. It is the most abundantly expressed P450 in human liver and it is known to metabolize more than 120 different drugs. Examples of CYP3A4 substrates relevant in human space flight include: acetaminophen, diazepam, erythromycin, lidocaine, lovastatin, and warfarin. CYP3A4 also is sensitive to enzyme induction, which tends to lower plasma concentrations of CYP3A4 substrates, resulting in reduced efficacy of the substrate.

Some CYP450 genes are highly polymorphic, resulting in enzyme variants that may shape variance in drug-metabolizing capacities among individuals at Earth gravity (1G), let alone the microgravity environment. For context, it is estimated that genetics account for 20–95 % of variability in drug disposition and effects (Hitchen 2006). CYP450 metabolic capacities may be described as follows (Tamási and Falus 2012).

Extensive metabolizers (also called Normal Metabolizers)

- have two active CYP450 enzyme gene alleles, resulting in an active enzyme molecule

Intermediate metabolizers

- have one active and one inactive CYP450 enzyme gene allele
- May require lower dosage than normal, though pro-drugs may require higher dose

Poor metabolizers

- lack active CYP450 enzyme gene alleles
- may suffer more adverse events at usual doses of active drugs due to reduced metabolism and increased concentrations
- may not respond to administered pro-drugs that must be converted by CYP450 enzymes into active metabolites

Ultrarapid metabolizers

- have 3 or more active CYP450 gene alleles
- may not reach therapeutic concentrations at usual, recommended doses of active drugs
- may suffer adverse events from pro-drugs that must be converted by CYP450 enzymes into active metabolites
- May require higher doses of pro-drugs

For space flight, a list can be generated identifying the CYP450 enzymes with genetic variants of a specific individual. This list can be cross-referenced with the particular mission drug list. When conflicts arise between the CYP SNP profile and the mission drug list, alternative drugs can be selected that are metabolized via a different CYP pathway.

The U.S. Food and Drug Administration (FDA) has already approved a microarray device that can detect 29 variations in *CYP2D6* and *CYP2C19* (Shizukuda et al. 2009). Follow-on multiplex assays for (*CYP2C19*) and (*CYP2D6*) have also been approved by the FDA. Cytochrome P450 profiles of selected drugs that might be found on a mission drug list are noted in Table 1.

**Table 1:** Substrates, inhibitors, and inducers of human CYP450s

	CYP1A2	CYP2B6	CYP2C9	CYP2C19	CYP2D6	CYP2E1	CYP3A4
Substrates	Caffeine	Bupropion	Diclofenac	Omeprazole	Bufuralol	Acetaminophen	Nifedipine
	Imipramine	Midazolam	Losarten	Phenytoin	Codeine	Ethanol	Erythromycin
	Tacrine	Tamoxifen	Phenytoin	Indomethacin	Desipramine	Chlorzoxazone	Midazolam
	Theophylline	Verapamil	Tolbutamide	R-warfarin	Lidocaine	Sevoflurane	Testosterone
	R-warfarin	Testosterone	S-warfarin				
Inhibitors	Ciprofloxacin	Ketoconazole	Fluconazole	Cimetidine	Quinidine	Disulfiram	Ketoconazole
	Furafylline	Tranylcypromine	Sulfaphenazole	Ketoconazole	Methadone		Erthyromycin
	Mibefradil	Troglitazone	Paroxetine	Paroxetine	Cimetidine		Grapefruit juice
	Ticlopidine	Orphenadrine		Ticlopidine	Fluoxetine		Ritonavir
Inducers	Insulin omeprazole (cruciferous vegetables) (char-grilled meat) (tobacco)	Dexamethasone Phenobarbital Rifampin Sodium valproate	Rifampin Secobarbital	Prednisone Rifampin	None identified	Ethanol (starvation)	Carbamazepine Phenobarbital Phenytoin Rifampin

A partial list of CYP450 inhibitors, substrates, and inducers. For a complete list, see (Flockhart 2007)

### Personalized drug regimens based on Phase II conjugation

For many drugs, metabolism via CYP450 is the first phase of drug biotransformation, as noted. These same drugs frequently pass through a Phase II biotransformation reaction. Phase II drug metabolism reactions are generally characterized as conjugation reactions, wherein a small molecule is bound to the drug metabolite to improve solubility for eventual excretion.

Characterization of individual astronaut Phase II profile might consider at least three features. These are: (1) *SNP variant profiles* of Phase II conjugation enzymes, (2) *adequacy of micronutrient cofactors* of Phase II enzymes, and (3) *adequacy of* *conjugation agents* that directly bind drugs, as part of Phase II conjugation.

Phase II profiles should be conducted for each astronaut and, where the evidence is sufficient, be used to develop appropriate countermeasures. For the Phase II profile, assessment considerations include, but are not limited to:

*SNP variant profiles of Phase II conjugation enzymes* For instance, UGT (UDP glucuronosyltransferases) is an enzymatic superfamily, which is involved in conjugation of endogenous compounds (bilirubin, steroidal hormones, thyroid hormones, biliary acids, vitamins) and exogenous compounds (drugs, carcinogens, polluting dietary elements) that are transformed in N-, O-, S-, C-glucuronates. They are responsible for roughly 35 % of Phase II reactions. Understanding genetic variants of Phase II enzymes will be helpful in designing individualized drug regimens (Crettol et al. 2010).

*Adequacy of nutrient cofactors of Phase II enzymes* For instance, riboflavin is a cofactor for glutathione reductase. Assess pre-mission status of all nutrient cofactors to ensure optimum status for each individual astronaut. If warranted by genotype, provide nutrient cofactor at dosage commensurate with the allelic variant (e.g. wild type, heterozygote, homozygote).

*Conjugation agents that directly bind drugs, as part of Phase II conjugation* Assess pre-mission status of key conjugation agents (e.g. glutathione, glycine, cysteine, glutamine, arginine, taurine, acetate) to ensure optimum status for that individual. For example, in most cases of glutathione conjugation, more polar glutathione conjugates are eliminated into the bile or are subsequently subjected to other metabolic steps. This eventually leads to formation of mercapturic acids, which are excreted in urine. In these cases, glutathione is depleted as it binds drugs through the conjugation of Phase I intermediate metabolites. This depletion can reduce available glutathione for future drug metabolism reactions and alter the redox balance of the cell.

For instance, the analgesic acetaminophen is converted to the electrophilic *N*-acetyl-*p*-benzoquinone imine (NAPQI), which is conjugated for removal by glutathione. If glutathione is in poor supply, NAPQI exerts highly toxic effects by covalent reactions with proteins, such as those found in mitochondria. This can lead to liver damage (Jaeschke and Bajt 2006).

If glutathione depletion is identified through blood chemistry (low GSH or low GSH:GSSG ratio), pre-mission glutathione or glutathione precursors can be provided at the dosage needed to assure optimum mission status. Stable glutathione precursors, such as *N*-acetylcysteine, can be provided on missions.

### Personalized drug regimens based on ethnicity

Ethnic differences in CYP450 isoforms, such as CYP2D6 and CYP2C19, can be pronounced and warrant consideration when embarking on long duration space missions. For instance, most western populations are characterized by roughly 93 % normal (or efficient) metabolizers, 7 % poor metabolizers, and 1 % ultra-rapid metabolizers of CYP2D6. In contrast, only 1 % of Asians are considered poor metabolizers of CYP2D6. Roughly 20 % of Asians are poor metabolizers via CYP2C19, while only about 4 % of Caucasians are considered poor metabolizers via this isoform (Jain 2009).

CYP3A5 expression also varies widely with ethnicity. For instance, more than 50 % of African Americans express CYP3A5, while some 30 % of Caucasians express this isoform. Understanding ethnic variance in CYP functionality has the potential to greatly enhance astronaut safety and performance, by assuring that prescribed drugs work as expected and that the probability of adverse reaction to these drugs is minimized.

### Omics and drug metabolism in space

Using pharmacogenomics and targeted metabolomics to personalize drug prescribing, is one preventive method to improve safety and efficacy of drugs used in space. Assessing specific gene, protein, and metabolite changes in *response* to drug administration, represents an application of Omics that can more thoroughly characterize off-target (or unanticipated) drug effects in space or space analogue environments.

## One carbon metabolism and human gene variants on earth and in space

**Fig. 2:**
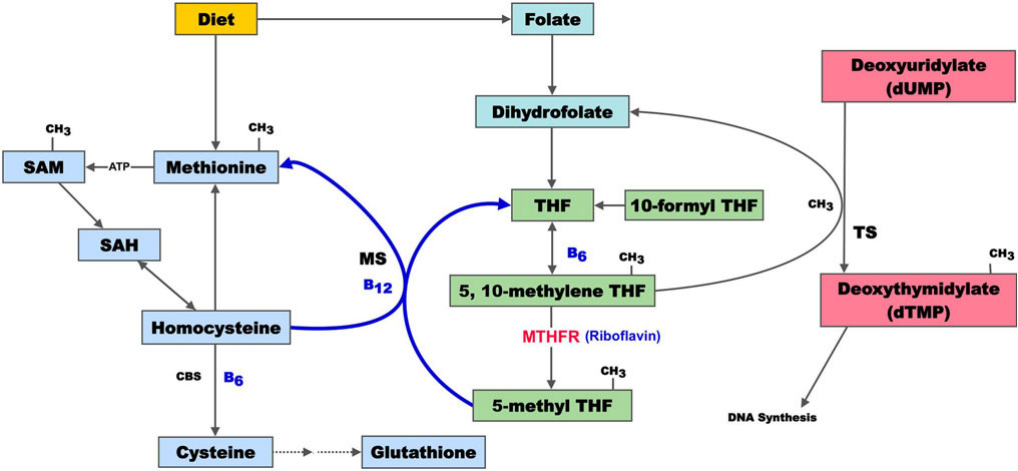
Summary of one carbon metabolism. Dietary methionine is converted to homocysteine (Hcy), cystathionine, and cysteine, which can be further converted to form glutathione. Inadequate B12, folate, or B6 may lead to elevated Hcy, with its attendant biological effects. Depleted glutathione may impair free radical defenses, as well as the ability to conjugate prescription drugs in space. Folate, in the form of 5,10-methylene THF, is involved in the transfer of one carbon units. 5,10-methylene THF is reduced to 5-methyl THF by MTHFR, which is the site of very common genetic polymorphisms. The methyl group is transferred to dUMP for dTMP synthesis, which is an irreversible rate-limiting step in DNA biosynthesis. Sustained disruption of one carbon metabolism results in misincorporation of uracil into the DNA backbone in place of thymine, a change that is equivalent to a mutational event. In general, disordered one carbon metabolism by genetic polymorphism, micronutrient deficit, or both can lead to several downstream effects with implications in space flight. These include (1) elevated Hcy, (2) decreased glutathione synthesis, and (3) unstable DNA (adapted from: Lamprecht and Lipkin 2003)

One carbon metabolism involves the transfer of methyl groups (CH3) from donors, such as folate, B12, choline, betaine, and trimethylglycine. Folate, B12, and choline are essential inputs that must be obtained from the diet. A deficit in methyl donors, leads to a series of adverse events important to humans in space. The enzymes that govern one carbon metabolism are produced from a series of methyl transfer genes. Common polymorphisms in key methyltransferase genes also lead to adverse events of importance. Frequently, convergence exists between altered micronutrient intake and genetic polymorphisms (Fig. 2). The potential implications for human space flight are only just emerging.

### One carbon metabolism and neuro-ocular health

There is mounting evidence that specific health risks, such as neuro-ocular changes [and increased intracranial pressure (ICP)], are related to disordered one-carbon metabolism involving folate, B12, and Hcy. Approximately 20 % of ISS crew members on space flight missions of 4 months or longer have experienced ocular changes and persistent visual problems on return to Earth. These were correlated with significantly elevated levels of Hcy, methylmalonic acid and cystathionine, along with reduced levels of folate and Vitamin B12 (Zwart et al. 2012). The metabolite data indicate that a significant proportion of crew members may have associated genetic traits that contribute to disturbed one-carbon metabolism. In addition, the metabolic pathway mutation described above has been conclusively linked to the production of oxidative stress and damage associated with coronary artery disease (Vijaya Lakshmi et al. 2011). While the link between space flight-induced ocular changes and altered one-carbon metabolism must be replicated in follow up studies, the existence of such a link may have significant implications, given the altered fluid dynamics known to occur in space flight.

Otto and colleagues have documented that 15 long-duration crewmembers have experienced in-flight and post flight visual and anatomical changes including optic-disc edema, globe flattening, choroidal folds, and hyperopic shifts as well as documented post flight elevated ICP. In the post flight time period, some individuals have experienced transient changes while others have experienced changes that are persisting with varying degrees of severity.

While the underlying etiology of these changes is unknown at this time, the NASA medical community suspects that the microgravity-induced cephalad-fluid shift and commensurate changes in physiology play a significant role (*NASA Evidence Report Risk of Spaceflight*-*Induced Intracranial Hypertension and Vision Alterations*) (Otto 2012; Mader et al. 2011). Furthermore, in retrospective examination of data, >60 % of long-duration crewmembers (ISS/MIR) and >25 % of short-duration (Shuttle) crewmembers have reported subjective degradation in vision (based on debrief comments) (Mader et al. 2012; Gibson et al. 2012). Decreased near-visual acuity was demonstrated in 46 % of ISS/Mir and 21 % of Shuttle crewmembers, resulting in a shift of up to 1–2 diopters in their refractive correction. It is also known that Hcy is a causative agent in retinal ganglion cell death (Ganapathy et al. 2011). These phenomena may have direct implications for the documented loss of vision that ISS astronauts and other long durations flyers have experienced.

### One carbon metabolism, chromosome instability, and space radiation

**Fig. 3:**
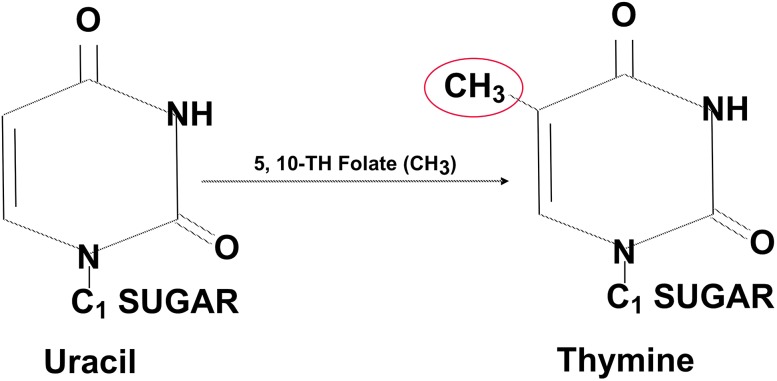
Methyl transfer, uracil, and thymine. A methyl group (–CH3) is transferred from folic acid to uracil in the formation of thymine. If methyl groups are limited in availability (via genotype, micronutrient deficit, or both), uracil accumulates in the nucleus

**Fig. 4:**
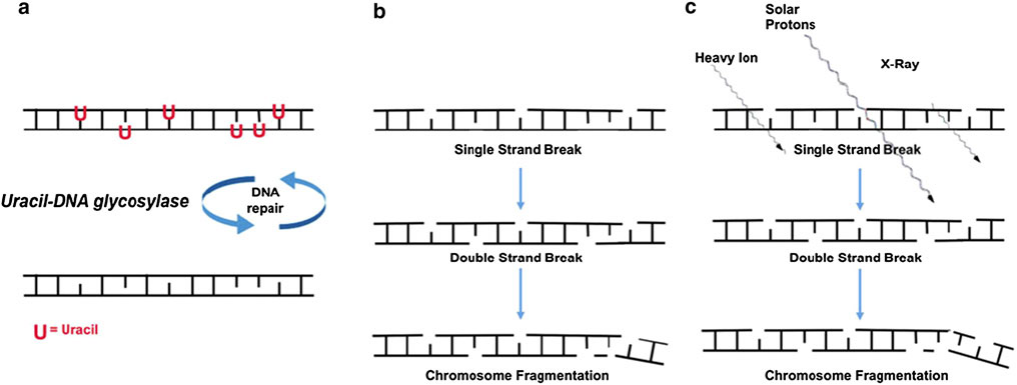
Uracil, unstable DNA, and radiation. **a** Methylation defects lead to high nuclear uracil. Incorporation of uracil (U) into DNA activates the uracil-DNA glycosylase repair system to repair the DNA strand. Repair glycosylase enzymes attempt to excise the uracil residues, which involves temporarily cleaving the DNA backbone. However, if the ratio of dUTP to dTTP is still elevated (due to high nuclear uracil), this re-synthesis may again incorporate uracil instead of thymine. **b** This cycle may lead to DNA single strand breaks, double strand breaks, and eventual chromosome fragmentation (equivalent to a mutational event). **c** Exposure of this unstable DNA to subsequent radiation in space (heavy ions, solar protons, x-rays, etc.) may initiate additional DNA damage (representing two convergent mutational events). Adapted from Békési, A 2004

A set of convergent variants in one carbon metabolism is known to strongly influence chromosome stability via direct effects on DNA. In nucleic acid synthesis, deoxythymidylate (dTMP) is synthesized from deoxyuridylate (dUMP), through one carbon transfer from a methyl donor. The methyl donor is typically methylfolate, though methyl transfer is also strongly influenced by B12 and choline status (choline metabolism intersects with folate metabolism at the methylation of Hcy to form methionine) (da Costa et al. 2006). When methyl groups are unavailable from folate, conversion of uracil to thymine is reduced. This results in an alteration of the dTMP/dUMP ratio and in uracil accumulation in cell nuclei (Ames 2000) (Figs. 3, 4).

Normally, when uracil appears in DNA, it is excised by uracil glycosylase enzymes. However, with excessive uracil accumulation, the process can lead to transient single-strand breaks in DNA. Two opposing single-strand breaks can lead to double-strand chromosome breaks, which are more difficult to repair and can lead to genome instability. Common fingerprints of such changes include micronuclei, nucleoplasmic bridges, and nuclear buds (Courtemanche et al. 2004).

In a small (*N* = 22) cross-sectional study of micronucleus frequency in erythrocytes of splenectomized subjects, Blount et al. showed that the elevated micronucleus index was strongly associated with low levels of serum folate. In this study, uracil levels were found to be 70 times higher in subjects whose serum folate <4 ng/ml (as high as 4 M uracil residues/cell). After 3 days supplementation with 5 mg folic acid, uracil levels were rapidly reduced. This was accompanied by corresponding reductions in erythrocyte micronucleus frequency, though this took much longer (Blount et al. 1997). Of interest is a finding that men with normal concentrations of folate and vitamin B12, but Hcy levels >10 mol/l had significantly increased micronucleus index (*p* = 0.05). This was in comparison to those with normal folate, normal vitamin B12, and Hcy <10 mol/l. Elevated Hcy status, in the absence of vitamin deficiency, and low, but not deficient, vitamin B12 status appear to be important risk factors for increased chromosome damage in lymphocytes (Fenech 2012).

Titenko–Holland conducted a folic acid depletion/repletion study (base-line: 195 μg/day; depletion: 5 weeks 65 μg/day; repletion: 4 weeks 111 μg/day followed by 20 days > 280 μg/day) in nine postmenopausal women in a metabolic unit. These women showed a significant increase in micronucleus frequency in lymphocytes following depletion and a decrease following repletion. Micronucleus frequency in buccal cells decreased after the repletion phase. The depletion phase in this study also resulted in DNA hypomethylation, increased dUTP/dTTP ratio, and lowered NAD levels in lymphocytes (Titenko-Holland et al. 1998).

Fenech et al. performed a randomized double-blind placebo-controlled dietary intervention study (*N* = 31, 32 per group) to determine the effect of folate and vitamin B12 (B12) on DNA damage (micronucleus formation and DNA methylation) and plasma Hcy in young Australian adults aged 18–32 years. The dietary intervention involved supplementation with 700 μg folic acid and 7 μg vitamin B12 in wheat bran cereal for 3 months, followed by 2,000 μg folic acid and 20 μg vitamin B12 via tablets for a further 3 months. This study revealed that micronucleated cell frequency is minimized when plasma Hcy is below 7.5 mol/l, serum B12 is above 300 pmol/l, and red cell folate is above 700 nmol/l. The study also revealed that elevated plasma Hcy may be a direct risk factor for chromosome damage (Fenech 1998, 2012).

Milić et al. studied 36 healthy males who were not vitamin deficient. They found a positive association between an increased number of micronuclei and lower plasma vitamin B12 concentrations, suggesting vitamin B12 levels are instrumental in maintaining DNA integrity—not just in vitamin B12 deficient population, but also in a healthy one (Milic et al. 2010). Kapiszewska et al. demonstrated that more than 400 pg/ml of vitamin B12 in plasma in subjects with a positive folate balance is critical for genomic stability, with uracil misincorporation into DNA being further related to the MTHFR genotype (Kapiszewska et al. 2005). The level of folate and B12 needed to prevent genome instability is significantly greater than the level needed to prevent anemia (Table 2).

**Table 2:** Estimated level of folate and B12 needed to prevent genome instability

	Prevent anemia	Minimize DNA damage
Plasma folate (ng/ml)	2.2	21 (7.3–53.0)
RBC folate (ng/ml)	132	313–700
Plasma B12 (pmol/l)	150	400

Beck and Olek (2003), Fenech (2012)

Variants in one carbon metabolism that induce uracil accumulation in DNA, defective DNA repair, and chromosome instability, may render folate-deficient cells more sensitive to the damaging effects of a second external stress (Ames 2000). This raises particular concerns for astronauts with methyl cycle defects who are exposed to ionizing radiation from a variety of sources, including galactic cosmic rays, solar protons, and high energy electrons and protons trapped by the Earth’s magnetic field (Van Allen Belts).

To put this in perspective, it is estimated that, during a mission to Mars, every cell nucleus in an astronaut’s body would be hit by a proton or a secondary electron every few days, and by an HZE particle about once a month (Jain et al. 2011). Recent data from the Radiation Assessment Detector (RAD) experiment on the Mars Science Laboratory (Zeitlin et al. 2013) reveal that a during a 360-day Mars transit mission, an astronaut would receive a dose of about 662 millisieverts (mSv). This is just short of international space agencies career exposure limits of 1,000 mSv, a limit that corresponds to a 3 % risk of exposure-induced death from cancer (Kerr 2013). Levels of exposure for sensitive neural tissue like the hippocampus are independently set by NASA at 500 mSv per year and 1,500 mSv for a career (Cucinotta et al. 2012).

These exposure data argue for a dedicated effort aimed at better characterization of all inherent influences on DNA stability and DNA repair prior to entering such radiation environments, as well as during such exposure.

This is highlighted by concern about the extent to which one carbon defects may actually mimic the degree of DNA damage one might encounter in the space radiation environment. For instance, Fenech et al. found that the chromosomal damage in cultured human lymphocytes, caused by reducing folate concentration from 120 to 12 nmol/l, is equivalent to that induced by an acute exposure to 0.2 Gy of low linear-energy-transfer ionizing radiation (e.g., X-rays), a dose of radiation that is 10 times greater than the annual allowed safety limit of exposure for the general population (Hartwig 2001; Fenech 2010; Fenech and Crott 2002).

Raising additional concern is the potential impact of one carbon deficits and concomitant exposure to radiation in space. While this has not been studied in space, Earth-based* ex vivo* and in vitro studies are informative. Fenech et al. studied the combined effect of folic acid deficiency and radiation exposure on genome stability, using cultured lymphocytes of 12 human subjects with different MTHFR genotypes. *Ex vivo* cells were grown for 9 days, using different concentrations of folic acid (12, 24, and 120 nM folic acid), and exposed to 0.5 Gy of gamma rays.

The effect of folic acid was highly significant (*p* < 0.001) and explained >50 % of variance of both types of micronuclei. Also, nucleoplasmic bridges and buds were significantly increased under low folate supply. The increase in bridges was mainly observed in MTHFR 677*TT* cells, highlighting a significant effect of the MTHFR genotype (*p* < 0.006) on this biomarker.

Beetstra et al. studied the micronucleus assay in 10-day WIL2-NS cell cultures at four different folic acid concentrations (0.2, 2, 20, and 200 nM) that span the physiological range in humans. Folic acid deficiency and γ-irradiation were shown to have a significant interactive effect on frequency of cells containing micronuclei, with the frequency of radiation-induced micronucleated cells increased with decreasing folic acid concentration (Beetstra et al. 2005). Exposure to 20 nM folate resulted in effects similar to those caused by 1.0 Gy of radiation, a level that is carcinogenic and some 50 times the annual safe radiation exposure upper limit. While both radiation and folate deficiency caused DNA breaks, their effect on gene expression were somewhat divergent. Radiation activated excision and DNA double-strand break repair genes, but it repressed mitochondrial genes. Folate deficiency activated base and nucleotide excision repair genes, but not DNA double strand break repair genes.

These studies make a compelling case for how one carbon dysregulation may trigger DNA effects similar to those seen in radiation exposure (representing a mutational event) and how concomitant exposure to space radiation (a convergent risk for mutational events) represents a set of conditions in need of attention by the space biomedicine community.

### One carbon metabolism and bone health

Loss of bone integrity is one of the most challenging aspects of space flight. For example, a group of astronauts who had spent from 4.3 to 6.5 months aboard the ISS showed an average loss of 14 % in femoral strength. In some subjects, the magnitudes of the reductions in proximal femoral strength were comparable to estimated lifetime losses associated with aging (Keyak et al. 2009, Smith and Heer 2002).

The effect of disordered one carbon metabolism on osteoclast activation, decreased osteoblast activity, and increased bone fragility renders it a compelling candidate for addressing this perplexing problem of space flight. Most evidence points to a dynamic interplay between Hcy (elevated), folic acid, vitamin B12, pyridoxine (decreased) (Dhonukshe-Rutten et al. 2005), and SNP variants, such as MTHFR (heterozygotes and homozygotes). In those with the MTHFR mutation, riboflavin [as a flavin adenine dinucleotide (FAD) cofactor] contributes further to the adverse effects of one carbon transfer derangement on bone.

In studies of cultured osteoclasts from healthy male donors (34 ± 5 years), the combined reduction of folate, B12, and B6 stimulated dentine resorption activity (DRA) up to 211 %. Reduction of only one of these vitamins stimulated DRA up to 250 % (folate: maximum increase 248 %, *p* < 0.018; B12: maximum increase 252 %, *p* < 0.001, B6: maximum increase 247 %, *p* < 0.001). In addition, Hcy in varying physiologic concentrations (up to 2,500 μmol/l) has been shown to stimulate dentine resorption up to 395 % (*p* < 0.001) (Jain 2009).

These findings are in agreement with human studies demonstrating a correlation between plasma concentrations of Hcy and biochemical bone resorption markers (Herrmann et al. 2005, 2007). A recent meta-analysis involving 14,863 subjects concluded that elevated Hcy significantly increases the risk of fracture (Yang et al. 2012).

Riboflavin deficit may confer additional risk to bone fragility, even in the absence of microgravity. For example, women homozygous for MTHFR 677*TT*, who were also in the lowest quartile of riboflavin intake, had a 1.8 (95 % CI 1.1–2.9, *p* = 0.01) times higher risk for incident osteoporotic fractures and a 2.6 (95 % CI 1.3–5.1, *p* = 0.01) times higher risk for fragility fractures compared with the 677*CC* genotype (interaction, *p* = 0.0002). In the lowest quartile of dietary riboflavin intake, *T*‐homozygous individuals (men and women combined) had higher (22.5 %) Hcy levels compared with *C*‐homozygotes (mean difference = 3.44 νM, *p* = 0.01; trend, *p* = 0.02) (Yazdanpanah et al. 2008).

The effect of one carbon derangement on bone in the astronaut population in space has not been investigated. Given that declining bone density is among the principal concerns surrounding humans in space, understanding the added risk of disordered one carbon metabolism warrants further attention.

### One carbon metabolism and hypertension

The conditions of increased ICP and intraocular pressure in space may also be partially linked to one carbon metabolism. Previously, the MTHFR *C*677*T* mutation has been shown to be a risk factor for hypertension in Earth-based conditions (Kesler et al. 2010). Riboflavin is an important essential input for those with the MTHFR mutation, since riboflavin is part of the FAD coenzyme complex that activates MTHFR. In vitro studies have shown that thermolabile MTHFR is ~10 times as likely as the wild-type enzyme to dissociate from its FAD prosthetic group and thus become inactivated (McNulty et al. 2002).

Wilson et al. examined a population of MTHFR 677TT homozygotes in 2004, with a follow up in 2008. They found a graded relation between the *T* allele and blood pressure at both time points, with a significantly higher systolic BP (by 16 mmHg) in patients with the *TT* genotype than in those with the *CC* genotype in 2004—a difference that remained significantly higher (by 13 mm Hg) in 2008. When riboflavin was given to *TT* homozygotes with elevated blood pressure, it produced an overall decrease in systolic (−9.2 ± 12.8 mmHg; *p* = 0.001) and diastolic (−6.0 ± 9.9 mmHg; *p* = 0.003) blood pressure. To put these results into an Earth-based context, it would take roughly 10 kg of weight loss or an exercise regimen that burned 4,200 kcal/week to achieve comparable decreases in blood pressure (Wilson et al. 2012).

Of further interest is the finding that individuals with the MTHFR mutation may suffer from deficits in spatial navigation. This has been found in MTHFR mutants with hypertension, which may be a result of the cerebrovascular changes associated with the variant (Deshmukh et al. 2009).

These findings argue in favor of assessing genotypes, such as MTHFR, in all astronaut training, with additional attention to the key essential input (riboflavin) that governs the MTHFR-FAD enzyme–coenzyme complex.

### One carbon metabolism and personalized countermeasures

To date, SNP variants in one carbon metabolism have not been characterized in the astronaut population. Given the findings of Zwart et al. regarding metabolites of one carbon metabolism and ocular disturbances on a subgroup of ISS astronauts, the influence of one carbon metabolism on blood pressure, the effect of one carbon metabolism on bone, the influence of one carbon metabolism on genome stability, and the potential additive effect of cosmic radiation on unstable DNA, it is evident that this single metabolic feature has potential far-reaching impact on humans in space.

As a further point of reference, our group routinely applies detailed molecular profiling on an individualized basis with human subjects entering extreme environments and in those participating in extreme competitive conditions. For example, we apply the personalized medicine paradigm to drivers in The 24 Hours of Le Mans racing event and in the US Le Mans Series (Heyman and Schmidt et al., unpublished results). With specific reference to variants in one carbon metabolism, we identified MTHFR heterozygosity (677*CT*) in four of six drivers (all of European heritage), which suggests that variance in one carbon metabolism may emerge in small subject populations similar to astronauts.

Fredriksen et al. profiled a large European population of 10,601 subjects for genetic polymorphisms related to one carbon metabolism. His group confirmed a high penetrance of both the MTHFR *C*677*T* and the *A*1298*C*, with only 14 % of subjects possessing the wild type for both SNPs, while 86 % possessed at least one mutant MTHFR allele. Fully 17.4 % were heterozygous for both MTHFR *C*677*T* and *A*1298*C* (Fredriksen et al. 2007) Table 3.

**Table 3:** Number of genotype combinations of polymorphisms in the MTHFR *C*677*T* and *A*1298*C* genes (adapted from Fredriksen et al. 2007)

	CC	CT	TT
MTHFR *C*677*T*			
1298*AA*	1,492	2,217	783
1298*AC*	2,469	1,848	16
1298*CC*	1,246	43	0

Under our proposed emergent model of personalized medicine for human space flight, one can envision that assessment of one carbon metabolic networks would become standard protocol for all astronauts. From this pre-mission assessment, medical teams may develop personalized countermeasures for each astronaut, regardless of where he or she falls on the continuum of these converging molecular influences.

A hypothetical pre-mission assessment of one carbon metabolic status is presented in Table 4. Countermeasures would be based on the status of individual biomarkers.

**Table 4:** Hypothetical pre-mission one carbon assessment

Markers of genomic stability	Single nucleotide polymorphisms	Markers of one carbon micronutrient status
Micronucleated lymphocytes	MTHFR (*C*677*T*, *A*1298*C*)	Hcy
Nuclear buds	MTR (*A*2756*G*)	
Nucleoplasmic bridges	MTRR (*A*66*G*)	Methylmalonic acid
		Folate
	BHMT (*G*742*A*)	B12
Total nuclear uracil	CBS (*C*699*T*)	Pyridoxine
	TCN2 (*C*776*G*)	Riboflavin

Assessment of the aggregate influences outlined in Table 4 would allow strategic preparation and countermeasure development for each individual, based on his particular genotype and metabotype, thus addressing, to the greatest degree possible, his molecular variances

*MTHFR* methylenetetrahydrofolate reductase, *MTR* methionine synthase, *MTRR* methionine synthase reductase, *BHMT* betaine Hcy methyltransferase, *CBS* cystathionine β-synthase, *TCN2* transcobalamin II (Adapted from Leopardi et al. 2006)

## Gene variants associated with iron metabolism on earth and in space

Gene variants contributing to disordered iron metabolism represent another example of metabolic alterations that may modify the risk profile of astronauts entering the space environment. Iron is the most redox-active trace element found in the human body. Interaction of bodily iron with gamma or other forms of radiation represents one level of risk. Exposure to changing oxygen saturation conditions in habitation versus extravehicular activities (EVA) conditions represents a hypoxia–hyperoxia paradigm, where the known presence of excess catalytic iron may pose additional risk. Beyond this, iron represents a potential general risk across all performance and disease conditions in space (Smith 2002a).

Iron exerts its primary oxidative effects via the Haber–Weiss and the Fenton reaction, producing highly reactive hydroxyl radical species. The Haber–Weiss reaction generates hydroxyl radical (·OH) from hydrogen peroxide (H_2_O_2_) and superoxide (·O_2_^−^).

The first step involves reduction of ferric ion to ferrous iron. Hydroxyl radicals formed from the Fenton reaction are strongly reactive with DNA, lipids, proteins, and a range of other biomolecules.

The Haber–Weiss reaction:

Fe3^+^ + ·O_2_^−^ → Fe2^+^ + O_2_

The second step is the Fenton reaction:

Fe2^+^ + H_2_O_2_ → Fe3^+^ + OH^−^ + ·OH

Net reaction:

·O_2_^−^ + H_2_O_2_ → ·OH + OH^−^ + O_2_

The unique role of iron as a potential risk factor in human space flight is highlighted by the high prevalence of the genetic variant for hemochromatosis (HFE). HFE is among the most common genetic variants in humans, with two common missense mutants: C282Y and H63D. Among Caucasians, one in 200–250 are homozygous for C282Y (Adams et al. 2005). The highest reported C282Y homozygosity was reported in Ireland (1/83) (Gleeson et al. 2004). In Caucasians, one in 50 are compound heterozygotes (one H63D and one C282Y mutant allele). As many as one in 8 to 10 are simple heterozygotes (carrier of one C282Y mutation).

In ~60–90 % of cases of HFE mutations, a missense mutation occurs at base 282, which causes tyrosine to be substituted for cysteine (C282Y). The tyrosine substitution results in dysregulation of transferrin-mediated uptake of iron in the gut. This leads to excess iron deposition in cells throughout the body, including but not limited to liver, heart, eyes, brain, joints, and pancreas (Jahanshad et al. 2012). While normal transferrin saturation is 45–55 %, saturation levels can rise to 65–85 % in those with this mutation. Normal serum ferritin levels are 50 ng/ml in wild type HFE homozygotes and may rise above 600 ng/ml in HFE mutant homozygotes.

### HFE, oxidative stress, and genome instability

The elevated iron burden encountered in HFE mutants has been shown to increase formation of 8-iso-PGF2α (a fingerprint of fatty acid oxidation) in Earth environments. Twenty-one patients with HFE (C282Y) were compared with 21 matched controls to determine whether HFE-related HFE was associated with increased oxidative stress.

Isoprostane levels in HFE subjects were determined to be 245 pg/mg creatinine (interquartile range 157–348) when compared with controls at 128 pg/mg creatinine (interquartile range 106–191; *p* = 0.002). After phlebotomy treatment and normalization of the iron parameters in the HFE group, 8-iso-PGF2α urinary excretion was lowered to control levels [146 (117–198) pg/mg creatinine, *p* = 0.38 vs. controls] (Kom et al. 2006).

Genetic material is uniquely susceptible to oxidative stress triggered by iron exposure. Common markers of nuclear RNA damage include 8-oxo-7,8-dihydroguanosine (8-oxoGuo), and related 2′-deoxyribonucleoside and ribonucleoside. In a study of newly diagnosed HFE patients, baseline 8-oxoGuo (RNA oxidation) was increased 2.5-fold over controls. Following phlebotomy (until serum ferritin was below 30 μg/l), 8-oxoGuo excretion returned to values similar to controls. Excretion of the DNA product 8-oxo-7,8-dihydro-2′-deoxyguanosine was reduced by 30 % following phlebotomy (Broedbaek et al. 2009).

This latter study demonstrates that oxidative stress is an important feature of the iron overload seen in hereditary HFE, which results in RNA oxidation that is reversible upon phlebotomy and strongly correlated with serum ferritin levels. Elevated iron and attendant elevation of 8-oxo-7,8-dihydro-2′-deoxyguanosine have also been linked with significant decreases in bone mineral density in the total hip, trochanter, femoral neck, and pelvis after space flight. **(**Zwart et al. 2013).

These oxidative changes associated with the HFE genotype may have cardiac implications for the asymptomatic subject. A recent study sought to examine the relationship between left ventricular diastolic function measured with tissue Doppler strain rate (SR; a sensitive echocardiography-derived measure of diastolic function), and oxidative stress in asymptomatic HFE subjects and control normal subjects (age 30–74). In the HFE subjects (confirmed *C*282*Y* homozygosity), the SR demonstrated moderate, but significant correlations with biomarkers of oxidative stress, with no correlations noted in normal subjects (Shizukuda et al. 2009).

Taken as a whole, the data suggest that the iron overload that accompanies HFE variants may contribute substantially to increased oxidative stress. Increased oxidative damage to nuclear material may have significant implications for the affected humans inhabiting space. The potential effect on cardiac function raises additional compelling reasons for understanding the genetic profile of space flight candidates (Hallgren and Sourander 1958; Nandar and Connor 2011). The convergent impact of radiation (gamma, proton and HZE particles, etc.) and iron on oxidized nucleic acids has not been studied to our knowledge.

### Hypoxia–hyperoxia paradigms associated with disordered iron metabolism

The finding of increased oxidative stress associated with HFE in Earthbound environments raises additional concerns when coupled with the unusual atmospheric conditions encountered in space flight. While iron is highly reactive under normal circumstances, the oxidative capacity of iron in the presence of high oxygen tension or oscillating oxygen levels in space has not been extensively studied. This is precisely the condition that will be encountered by astronauts engaged in prolonged missions BLEO to the Moon, Near Earth Objects, and Mars, because of the highly variable oxygen conditions deployed under the oxygenation and pressurization criteria outlined by current design reference missions (DRMs).

These DRMs result in conditions during EVAs, and in the exploration and habitat environments, that will expose astronauts to oscillations between alveolar hyperoxia (133 mmHg P_A_O_2_) and alveolar hypoxia (83 mmHg P_A_O_2_), respectively. Furthermore, oscillating arterial O_2_ (128 and 78 P_a_O_2_ mmHg) concentrations may be even more deleterious to the crew in light of the degradation in visual acuity data accumulated to date (Mader et al. 2011; Scheuring et al. 2007) (Table 5).

**Table 5:** Nominal oxygen partial pressures in next generation spacecraft and environments

Environment	P_B_		F_I_O_2_ (mmHg)	pO_2_ (mmHg)	P_I_O_2_ (mmHg)	P_A_O_2_ (mmHg)	P_a_O_2_ (mmHg)
	(psi)	(mmHg)					
MPCV	10.2	527	0.265	139	127	82	77
MMSEV	8.2	414	0.34	132	117	83	78
HABITAT	7.6	393	0.32	125	111	66	61
SUIT	4.3	222	1.0	222	175	133	128
Earth, Sea level	14.7	760	0.21	159	150	104	99

Comparative total pressures (PB), oxygen fractions (FIO2) and partial pressures of oxygen at various stages in the different environments of the Exploration vehicles (Courtesy of Dr. Johnny Conkin, NASA/USRA)

pO_2_ is ambient oxygen partial pressure; P_B_ × F_I_O_2_; FiO_2_ is the oxygen fraction; P_B_ is the total pressure; PIO_2_ is inspired oxygen partial pressure; (P_B_−47) × F_I_O_2_; P_A_O_2_ is alveolar oxygen partial pressure; (P_B_−47) × F_I_O_2_ − P_A_CO_2_ × (F_I_O_2_ + [(1−F_I_O_2_)/RER)], where P_A_CO_2_ is 40 mmHg and RER is 0.85; P_a_O_2_ is arterial oxygen partial pressure; P_a_O_2_ = P_A_O_2_ − 5 for the contribution of venous mixture

**Fig. 5:**
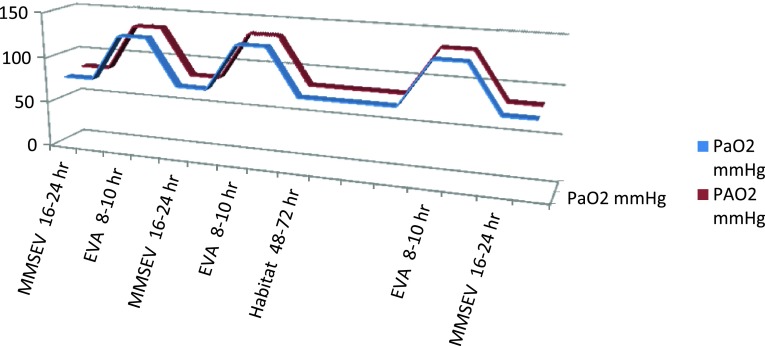
Cyclical variations in P_a_O_2_ and P_A_O_2_ in a typical habitat to EVA condition. A likely DRM: MMSEV at mild hypoxia for 16 of 24 h, then 8–10 h of EVA at hyperoxia. The EVA cycle would be every 48 h for any given astronaut, so it would be MMSEV 16 then 8–10 EVA; then 24 MMSEV, then 8–10 EVA and 16 MMSEV; then 24 MMSEV; then 8–10 EVA and 16 MMSEV. Then 48–72 h weekend off at Habitat/or MMSEV atmospheric conditions

The space suit used for EVAs will be a low pressure suit at ~4.3 psi and 90–100 % O_2_. Operations in the transit exploration environment are planned to be carried out at 7.6–8.2 psi and 34 % O_2_ in the habitat and multi-mission space exploration vehicle (MMSEV), and at 10.2 psi and 26.5 % O_2_ in the multipurpose crew vehicle (MPCV) (Conkin and Wessel 2008, 2009, Conkin 2011). DRMs for exploration environments in the next generation of U.S. space vehicles and habitats are summarized in Fig. 5.

The scientific literature reveals several potential problems with not only hypoxia (8.2/34), but also with hyperoxia (85–100 % O_2_). In combination, especially in an oscillating mode where hypoxia and hyperoxia are exchanged every 48 h during EVA/IVAs (Fig. 5), the P_i_O_2_, P_A_O_2_, and P_a_O_2_ values represent unknown risks under the proposed DRM scenarios.

No space exploration or habitation analogue study has yet employed this hypoxia–hyperoxia regimen. Thus, the only baseline data obtained derive from studies conducted on single events alone (i.e. hyperoxia or hypoxia) (Bitterman 2009), with these studies yielding data that indicate degenerative effects on the retina at high 90–100 % O_2_ concentrations (Xu et al. 2010). Combining significant delta O_2_ (hyperoxia/hypoxia) concentrations and pressures, which cause the retina to experience varying O_2_ concentrations, with the already problematic visual detriment effect, could lead to even greater visual acuity and recovery difficulties. This is anticipated to be accentuated during long duration missions, as high concentrations of O_2_ have been shown to be toxic in their own right.

Adding excessive total body iron to this hypoxia–hyperoxia paradigm has not been studied. However, the known high reactivity of iron in an oxygen-rich environment is expected to add an additional level of risk to those astronauts either heterozygous or homozygous for HFE, who have not had their iron status properly regulated. This is especially true for oxidant-sensitive tissues. For instance, it is known that HFE is associated not only with excessive accumulation of free iron in the retina and retinal pigmented epithelium, but also with excessive accumulation of heme. Since heme is toxic at high levels, as is free iron, heme-induced oxidative damage may also play a role in HFE-associated retinal pathology (Gnana-Prakasam et al. 2011).

In space, there is an additional stressor that may interact with the high iron genotype and the hypoxia–hyperoxia paradigm. This is high energy particle radiation (primarily proton energy in Low Earth Orbit and galactic cosmic radiation in BLEO), which has been extensively studied with regard to its impact on the lens (Cucinotta et al. 2001; Rabin et al. 2005; Jones et al. 2007). The scarce evidence on the effects of space flight on the retina is limited to a very few experiments on rats which showed retinal cell death due to high energy-particle radiation (Philpott et al. 1978, 1980). One recent project aboard the ISS (ALTEA) has been addressing functional effects of microgravity and cosmic radiation, most often reflected as abnormal visual perception (“light flashes”) (Narici et al. 2004). Combining hypoxia and hyperoxia with increased radiation exposure in the range that will be experienced by space travel and habitation BLEO has been recently shown to have pronounced and negative synergistic effects (Pietrofesa et al. 2013; NASA LSDA database 2013).

Space medicine physicians will be asked to mitigate the risk associated with the combinatorial events of reduced gravity, hypoxia–hyperoxia, and radiation. Understanding astronaut status with regard to body burden of catalytic metals, such as iron, should become a central component of this aspect of risk management.

### Iron metabolism in space flight

**Fig. 6:**
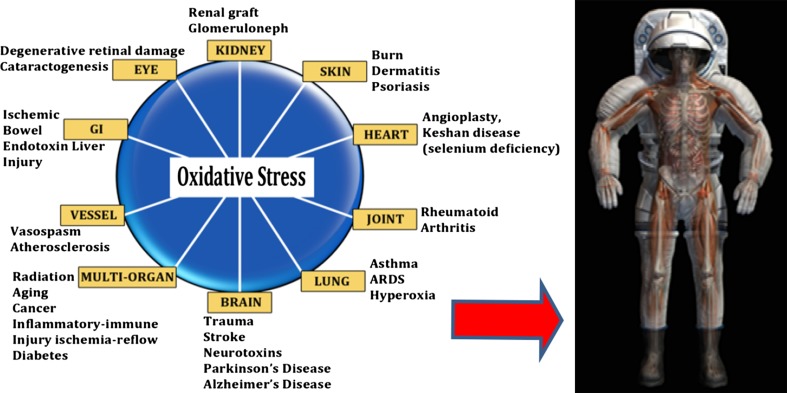
(**a**) Adapted from the National Institute of Standards and Technology; (**b**) Courtesy of NASA Outreach and Education

Iron metabolism in space flight is substantially different than on Earth, due to a host of physiologic anomalies peculiar to reduced gravity environments. Figure 6a and b illustrate many of the conditions experienced by humans on Earth, with similar conditions seen in astronauts in flight. Specifically relevant are changes in the health of the eyes, vessels, heart, lung, bone, immune system, and various organs.

Smith et al. have shown that iron metabolism is altered in astronaut populations, which is partially related to the food system and space conditions. In one space flight study, crew members consumed a mean of only 80 % of their recommended energy intake. On landing day, their body weight was less (*p* = 0.051) than before flight. This was associated with increased serum ferritin (*p* < 0.05), along with decreased ferritin saturation and hematocrit. Other acute-phase proteins were unchanged after flight, suggesting that the changes in iron metabolism are not likely to be solely a result of an inflammatory response. Collectively, these data suggest that storage pools of iron may be altered in the microgravity environment (Smith 2002a). Genotype was not profiled in these astronauts, but HFE heterozygosity or homozygosity would be expected to confer an additive risk of unknown proportion.

Also in this study, urinary 8-hydroxy-2′-deoxyguanosine concentration was greater, suggesting DNA damage was greater after the flight. RBC superoxide dismutase was less after flight (*p* < 0.05), further indicating increased oxidative damage. Despite vitamin D supplementation during flight, serum 25-hydroxycholecalciferol was decreased after flight (*p* < 0.01). Bone resorption was increased after flight, as indicated by several markers. Bone formation, assessed by several markers, did not consistently rise 1 day after landing. These data provide evidence that bone loss, altered vitamin D status, and oxidative damage are among critical nutritional concerns for long-duration space travelers. (Smith et al. 2001, 2005a, b).

### HFE and personalized countermeasures

To date, HFE genotypes have not been characterized in the astronaut population. Applying the personalized medicine paradigm, one can envision two hypothetical astronauts preparing to enter the space environment, each with distinctly different profiles with regard to iron status (Tables 6, 7). One is an astronaut with a normal (or wild type) HFE profile. She is a 35-year-old menstruating female, who loses blood (and iron) regularly as a result of menses. Her transferrin saturation is 45 % and her serum ferritin is 50 ng/ml (μg/l). Her mission team member is a 55 year old male astronaut who is homozygous (possesses two mutant alleles) for HFE. His transferrin saturation is 75 % and his serum ferritin is 600 ng/ml.

**Table 6:** Hypothetical personalized countermeasures based on genotype and metabotype

Gender	HFE Genotype (C282Y or H63D)	Serum ferritin	Transferrin saturation	Iron in food Iron supplementation
Female: 35 year old	Wild type (normal)	50 ng/ml	45 %	Iron in food permitted
				Yes, 18 mg/day suppl
Male: 55 year old	Homozygous (HFE)	600 ng/ml	75 %	Dietary iron restriction No iron supplementation Employ pre-mission phlebotomy to target serum ferritin at 50–100 ng/ml Monitor serum ferritin on long missions

**Table 7:** Suggested pre-mission iron assessment

Single nucleotide polymorphisms	Serum markers of iron status
HFE (C282Y), HFE (H63D)	Serum iron
	Serum ferritin
	Transferrin saturation
	Total iron binding capacity

At first glance, it is evident that the iron burden on the male astronaut is considerably greater than his female cohort. He is entering the high radiation space environment and cyclic hypoxia–hyperoxia conditions with a body burdened by high levels of a redox-active transition metal (Fe). We assert that this poses a unique risk that requires countermeasures tailored to his particular needs.

Under this scenario, the female astronaut will be prescribed a normal diet with regard to iron, while being supplemented with iron at a typical dose of 18 mg/day. The male will be prescribed a heme–iron-restricted diet with no iron supplementation. In addition, he should undergo a series of pre-mission phlebotomy treatments for a period of several months, until his serum ferritin is lowered to ~50–100 ng/ml (the exact target of which the aerospace medical community should address by some consensus). For extended missions, a policy will have to be developed to monitor and manage serum iron while in space craft or in habitats.

In this section, we explored the impact of selected genotypes on potential aspects of astronaut health and performance by citing only three categorical examples involving SNPs of relatively high penetrance, involving: (1) drug metabolism, (2) one carbon metabolism, and (3) iron regulation. It is important to note that some 3–10 million SNPs exist in the human genome (Human Genome Project Database). The space biomedicine field employing personalized medicine will be expected to narrow this list to those SNPs with the greatest relevance to humans entering the uniquely extreme environment of space. Those SNPs associated with the following phenotypes would be considered among those of initial import including, but not limited to, (1) drug responsiveness, (2) disease susceptibility, (3) performance deterioration, (4) physical tolerance, (5) psychological tolerance, (6) muscle metabolism, and (7) bone metabolism. Thought leaders in the field of personalized medicine and human space flight will have to consider those likely to be most responsive to countermeasures.

## Small molecules, essential inputs, DNA repair, and metabolic networks on earth and in space

In the previous section, we described three examples of genetic variants in a manner that represents a novel way of looking at individual astronaut susceptibility, risk, and countermeasure development. In each case of the cited examples, there are key small molecules (or elements) that strongly modify the effect these gene variants will likely have on the human response in space. We collectively refer to these small molecules and elements as *essential inputs.*

*Essential inputs* refers collectively to the class of small molecules, amino acids, vitamins, fatty acids, and trace elements that must be obtained from the diet. They cannot be synthesized by the human body. This also includes *conditionally essential* *inputs* that may become essential, as a result of genotype, drug therapeutics, environmental conditions, or dietary deficiencies of essential precursors. They are collectively referred to as *inputs*, because these are external components that input into the system to collectively influence all metabolic activities in humans. These effects can be pronounced, regardless of the genotype of the individual.

It is tempting to view these micronutrient essential inputs only in general ways and to underestimate the impact of relative states of insufficiency. However, if one gives the requisite attention to the core metabolic steps in which these elements participate, the true magnitude of small deficits in these inputs can be appreciated.

As a point of reference, a flavin-containing cofactor, FAD or FMN (riboflavin-derived), is utilized by 151 (4 %) of the 3,870 enzymes catalogued in the ENZYME database. Pyridoxal-5-phosphate (vitamin B6) is utilized by 112 (3 %) of the 3,870 enzymes catalogued in the ENZYME database (Ames et al. 2002).

Niacin is involved in over 400 NAD(P)-dependent reactions, giving it the potential to influence every area of metabolism, including genomic stability, impaired cell cycle arrest and apoptosis, delayed DNA excision repair, accumulation of single and double strand breaks, chromosomal breakage, and telomere erosion (Kirkland 2012). The role of nicotinamide (niacin) in DNA repair and maintenance of genomic stability is tightly related to its functions as an NAD + precursor and a substrate for PARP-1. PARP- 1 is a nuclear enzyme that detects DNA damage, binds to DNA single or double strand breaks, and then uses NAD + as a substrate to form nicotinamide and ADPribose (Surjana et al. 2010).

Zinc is a component of more than 3,000 zinc-associated transcription factors, including DNA-binding proteins with zinc fingers, and more than 300 enzymes, including several proteins involved in DNA transcription, regulation, and repair (e.g. DNA polymerase). For instance, PARP-1 has two zinc-fingers that bind to DNA strand breaks, causing catalytic activation, which leads to NAD^+^ consumption and poly (ADP-ribose) formation (Kirkland 2012). Zinc also plays an important role in protecting DNA from damage, as part of antioxidant complexes, such as Cu/Zn-superoxide dismutase. DNA base excision repair is a major pathway responsible for the repair of both cellular alkylation and oxidative DNA damage. One critical step in this pathway involves the cleavage of damaged sites in DNA by apyrimidic endonuclease (APE), an important endonuclease in base excision repair. Low cellular zinc increases the expression of (APE), most likely in response to DNA damage induced by low zinc (Ho 2004).

Selenium is involved in a smaller total number of enzyme systems. However its influence is vast. For instance, three different selenium-dependent iodothyronine deiodinases (types I, II, and III) can both activate and inactivate thyroid hormone, by acting on T_3_, T_4_, or other thyroid hormone metabolites (Bianco and Larsen 2006). Selenium may also be protective by preventing DNA damage from occurring, by increasing the activity of repair enzymes, such as DNA glycosylases and DNA damage repair pathways that involve p53, BRCA1, and Gadd45 (Bera et al. 2013).

Varying, but similar “participation numbers” for a wide range of elements and small molecules can be observed for over 30 essential inputs, affecting molecular dynamics across almost all metabolic networks. Individually, deficits in one essential input have potentially significant effects on metabolic network regulation in humans in space. When deficits of multiple essential inputs converge, the effect on metabolic networks can be expected to be amplified.

### Essential inputs: magnesium (Mg), DNA repair, and ATP regulation in space

Mg is an appropriate example of an essential input by virtue of its participation in some 300 separate enzyme systems. The relevance of Mg is further highlighted by recent findings from the ISS, in which urinary Mg levels were found to be 44 % lower after landing than before launch (*p* < 0.001). Specifically, 55 % of ISS crew members had Mg concentrations lower than the low end of the clinical range (3.0 mmol/day) (Smith et al. 2005a, b). After 6 months in space, there is a loss of Mg reservoirs, with 35 % loss in some leg muscles (Fitts et al. 2010) and up to 2 % bone loss per month (Rowe 2010).

### Mg and DNA repair

Mg is an essential cofactor in almost all enzymatic systems involved in DNA processing, with a stabilizing effect on DNA and chromatin structure (Hartwig 2001). At its core, Mg cations bind to DNA and reduce the negative charge density, thereby stabilizing the structure of DNA (Anastassopoulou and Theophanides 2002). Mg is an essential cofactor for enzyme systems involving DNA repair, such as mismatch repair (MMR), base excision repair, and nucleotide excision repair (Hartwig 2001).

DNA MMR is responsible for the recognition and repair of mispaired bases and small insertion–deletion loops that are formed during DNA replication or recombination between non-identical DNA sequences. MMR activity lowers the mutation frequency in the genome by 2–3 orders of magnitude, with a loss of function of one of the MMR proteins resulting in a mutator phenotype. Mg influences switching in MutS (mismatch recognition enzyme) by inducing faster and tighter ATP binding, allowing rapid downstream responses. Mg is also essential for double-strand break repair, such as that encountered in one carbon deficits noted previously (Nishino and Morikawa 2002).

Mg status may have significant ramifications for astronauts entering conditions where demands on DNA repair are increased through radiation exposure and oxidative stress. For instance, low dietary Mg intake has been associated with poorer DNA repair capacity and increased risk of some cancers. In joint effects analyses, compared with those with high dietary Mg intake and proficient DNA repair capacity (DRC), the OR (95 % CI) for lung cancer in the presence of both low dietary Mg and suboptimal DRC was 2.36 (1.83–3.04) (Mahabir et al. 2008).

### Mg and energy metabolism

Maintaining adequate or optimal Mg status in the space environment may also be necessary to optimize work capacity and regulate oxygen utilization. This is based on the central role of Mg in energy metabolism. For instance, roughly one-third of all intracellular Mg is found within mitochondria. Mg bound to ATP is the sole biologically active form of ATP found in humans (wherein Mg is bound to the phosphate groups of ADP and ATP, as MgATP^2−^). Moreover, Mg is a central ion in ATP synthase, binding to phosphate in the catalytic F_1_ moiety of ATP synthase. Also, Mg is required for a series of enzymes involved in glycolysis (Table 8), adding further to Mg’s central role in energy metabolism.

**Table 8:** Magnesium effects across metabolic networks

Enzyme	Pathway	Function
Hexokinase	Glycolysis	Glucose to glucose-6-phosphate
Phosphofructokinase	Glycolysis	Fructose-6-phosphate to F-1,6-bisphosphate
Phosphoglycerate Kinase	Glycolysis	1,3-Bisphosphoglycerate to 3-phosphoglycerate
Enolase	Glycolysis	2-Phosphoglycerate to phosphoenolpyruvate (PEP)
Pyruvate kinase	Glycolysis	PEP to enolpyruvate
Isocitrate dehydrogenase	TCA cycle	Isocitrate to α-ketoglutarate
Phosphoenolpyruvate carboxykinase	TCA cycle	Oxaloacetate to PEP
ATP synthase	Electron transport chain	Magnesium plays a pivotal role in formation of the transition state where ATP is synthesized from ADP and inorganic phosphate
ATP stability	All ATP reactions	Mg-ATP is the physiologically active form of ATP

Magnesium is involved in metabolic pathways across vast networks, such as glycolysis, the citric acid cycle, and ATP regulation. Genomics, transcriptomics, proteomics, and metabolomics afford the ability to detect shifts in such networks, which can be used to inform countermeasure development

**Fig. 7:**
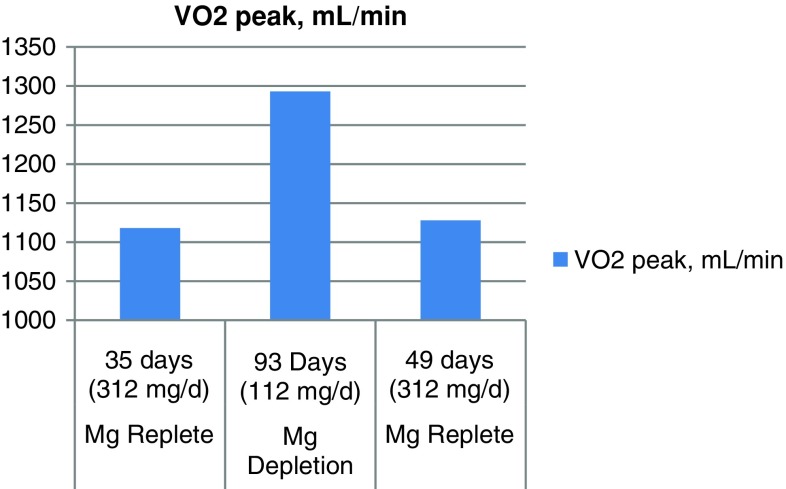
Oxygen use during Mg depletion and repletion. During Mg depletion, the amount of oxygen required to do a given amount of work is increased, thus reflecting decreased aerobic capacity efficiency during Mg deficiency. Values for Total VO_2_ and cumulative net oxygen uptake were consistent with these conclusions derived for VO_2_ peak values (adapted from Lukaski and Nielsen 2002)

Depletion–repletion studies involving Mg demonstrate that efficiency of oxygen utilization may be substantially altered during Mg depletion. Specifically, when Mg levels were low (112 mg/day), the total amount of oxygen required for a given amount of work was increased. When Mg levels were raised (312 mg/day), the same amount of work required less oxygen (Lukaski and Nielsen 2002). These findings correlated with increased RBC and muscle Mg (replete) and decreased RBC and muscle Mg (depletion) (Fig. 7). While this has potential implications for astronaut safety and performance, it may also have implications for engineering related to oxygen provision in habitats or within space suits.

If the effects of Mg on VO_2_ persist in the space environment, one must view the declining Mg status encountered with mission duration in a new light. If Mg status declines with mission duration, especially on the very long duration missions envisioned for Mars, then there is a hypothetical impact of mission duration on VO_2_ max, energy efficiency, and potentially also on DNA stability.

## Omics technology approaches: combinatorial influences complicate strategies for countermeasure development

The need for a systems biology approach to develop personalized medicine programs for astronauts derives from two key convergent influences. First, there are vast molecular networks that interact dynamically to influence astronaut *susceptibility* to any specific environment or condition to which he or she is exposed. Second, there are a series of *mission stressors* that impact heavily upon the individual susceptibility of each astronaut. Whether one thrives within the space environment may be heavily dependent upon individual susceptibility, space environmental exposures, and whether the countermeasures deployed for an individual astronaut are sufficient to overcome these susceptibilities. By illustration, one can use DNA stability to explore these convergent influences.

### Convergent effects of essential input deficits on DNA in space flight

DNA stability and DNA damage are among the key safety considerations in human space flight. Thus, we will use DNA stability here to establish a further premise that concomitant genetic variants and essential input deficits can aggregate to raise the risk of developing unstable DNA. On one hand, we have summarized data showing that one carbon deficits can lead to uracil accumulation in the nucleus, which may be followed by single- and double-strand DNA breaks. This alone represents a novel, but modifiable risk to DNA damage in space.

A further influence described herein is that of iron excess, which is associated with a common single nucleotide polymorphism (HFE). As noted previously, elevated iron can lead to the oxidation of DNA and RNA, adding yet another potential convergent variable to the space flight equation. A separate influence described herein is that of Mg, which is central to the activity of a wide range of DNA repair enzymes (base excision repair, MMR, etc.). As noted above, insufficient Mg has been associated with inefficient DNA repair, representing another novel, but modifiable risk to DNA instability in space.

One can quickly see the risk of a single gene variant or essential input deficit on DNA stability. The aggregation of two or more variants or essential input deficits represents an additional risk, which has not been properly characterized in astronauts. Under the limited scenario just described, one can envision a condition where altered one carbon metabolism (due to genetic variant and/or micronutrient deficit) leads to unstable DNA and DNA strand breaks, while a convergent deficit (Mg) leads to a reduced efficiency to repair such damage.

The phenomenon of two or more essential input deficits is plausible, given that one carbon deficits, declining Mg status, and altered iron metabolism have already been shown to exist within the flying astronaut population. But it also illustrates a greater premise asserted within this review. That is, there is a need to more broadly profile genotype, essential inputs, and metabolic networks in all astronauts, so as to begin to identify “off-target” influences that may bear upon individual susceptibility to the space environmental condition. This necessarily leads us to Omics approaches, which allow us to address the requisite complexity encountered in such analyses.

### Translational Omics strategy for space

The purpose of applying broad analytics and personalized medicine in human space flight participants is not to reduce participation or to find biomarkers that will limit the ability of astronauts to participate in missions. The focus of personalized medicine in human space flight is to develop countermeasures that are individualized to each space participant. The goal of this individualized approach is aimed at improving all aspects of mission performance, as well as limiting adverse sequelae of prolonged space flight. The emergent participation of space tourists will add yet another layer of complexity that can be addressed by personalization.

In this review, we primarily highlighted four areas of biological variance that are fundamental to a personalized medicine paradigm in human space flight. These include: (1) gene and small molecule variants associated with metabolism of therapeutic drugs used in space; (2) gene and small molecule variants associated with one carbon metabolism; (3) gene and small molecule variants associated with iron metabolism and oxidative stress; and (4) Mg as an essential input with effects on DNA stability and energy.

We have drawn attention to these areas for the purpose of simplicity. In practice, the same premise described in this review can be applied across a wide range of molecular processes. We suggest here that the more broadly we understand this variability of molecular networks in each astronaut, the better we will be able to develop countermeasures that optimize astronaut safety and performance.

This variability can be assessed by the application of Omics approaches to space medicine *research* (Table 9). For example, one can use Omics to better characterize the molecular events associated with the variable oxygen conditions that will be encountered in the space suit EVA condition *compared with* the space habitat environment noted previously (Figure 5). This can be done by using genomics (SNP profiling) to examine how genotype may be linked to tolerance in the two conditions. Transcriptomics, proteomics, and metabolomics can be used to further characterize the changing molecular dynamics that might be associated with the varying PAO_2_/PaO_2_/CO_2_ conditions.

**Table 9:** Proposed use of Omics in human space research

	Analogues	Pre-flight	In-flight	Habitation	Post-flight
Genome (targeted, non-targeted)	Yes	Yes	Yes	Yes	Yes
Transcriptome (targeted, non-targeted)	Yes	Yes	Yes	Yes	Yes
Proteome (targeted)	Yes	Yes	Yes	Yes	Yes
Metabolome (targeted, non-targeted)	Yes	Yes	Yes	Yes	Yes

Targeted and non-targeted Omics can be used as research tools in any space-related condition for the purpose of understanding human biological variance associated with space flight or space flight analogue conditions. These methods can be used to generate new hypotheses, serve as a basis for subsequent targeted analyses, and to identify appropriate molecular targets for subsequent use in developing personalized countermeasures. Note: The above also includes the trace element pool (sometimes called the metallome)

Patterns derived from these research-based Omics methods, can then be used to identify novel and relevant biomarkers, which may subsequently be used to develop *personalized countermeasures* applied to the individual astronaut entering such variable oxygen conditions (Table 10)

**Table 10:** Proposed use of targeted Omics as a basis for personalized countermeasure development

	Pre-flight	In-flight	Habitation	Post-flight
Genome (targeted)	Yes	No	No	No
Proteome (targeted)	Yes	Yes	Yes	Yes
Metabolome (targeted)	Yes	Yes	Yes	Yes

Targeted Omics consists of assessing pre-selected molecular markers. These biomarkers can be used to identify individual patterns that space medicine physicians will use in developing personalized countermeasures. A subset of selected biomarkers may warrant in-flight or in-habitat measurement, to assure that status for a particular mission objective is maintained or that specific safety thresholds are not exceeded. Note: The above also includes the trace element pool (sometimes called the metallome)

Introducing Omics into space biomedicine research adds a layer of complexity that will initially offer challenges in technology and methodology. But the ability to identify novel patterns, novel solutions, and predictive capability is expected to ultimately reduce complexity, by refining our engineering designs and human countermeasure approaches.

### Omics technology approaches to human space flight countermeasures

Earth-based human space flight research will benefit from the same range of technologies applied in all domains of systems biology. This includes, but is not limited to, LC–MS, GC–MS, NMR, ELISA, electrophoresis, PCR, gene arrays, protein arrays, flow cytometry, microscopy, and many others. These technologies will support assessment of genome, transcriptome, proteome, and metabolome in: (1) Earth-based space analogue research, (2) short-duration flight research where specimens are retained for ground analysis, (3) in-habitat research (such as the ISS) where retained specimens are returned to Earth for analysis, and (4) post-mission research.

These technologies can serve the purposes of human space flight research today, as well as providing the kind of analytics from which personalized countermeasures can be developed. However, real time Omics assessment during space flight and habitation presents a unique set of challenges, due to vehicle size, instrument weight, fluid handling characteristics in microgravity, and power constraints.

None of the current analytical technologies or methods is presently used by any space program to survey the changing molecular dynamics of astronauts in space. Instead, samples (blood, urine, or saliva) are frozen at −80 °C in the ISS (MELFI Rack freezers) and usually (every 90–180 days, notwithstanding schedule changes) transported to Earth for retrospective analyses, using some of the aforementioned technologies. Transport volumes are low, since the returning vehicles, such as the SpaceX Dragon and Soyuz vehicles, have limited cargo capacity, when compared with the retired Space Shuttle. Thus, multiple trips are required to transport MELFI freezer volumes to the ground.

This impacts research, as well as real-time assessment of markers relevant to astronaut health and safety. To further complicate the situation, exploration class missions beyond LEO to Moon and Mars will be constrained by cost and use smaller vehicle volumes than the ~400 m^3^ of habitable volume of the ISS (Wikipedia ISS and assembly pages; NASA Facts and Figs 2012). The smaller vehicles present two significant challenges to biochemical analyses. First, the vehicles will be roughly < 50 m^3^ in total volume, significantly limiting the potential size of the analytical equipment. Second, this reduced volume eliminates the ability to use the size- and power-hungry electrical systems, like the ISS MELFI freezers. This reduces the ability to store large amounts of samples for later analyses upon return to Earth.

Future in-flight and in-habitat analytical systems will require compact analytical solutions to transmit data to Earth, allowing for monitoring and intervention. The most likely and reasonable approaches to real time monitoring in these exploration vehicles will be, at least initially, proteomics-based, compact (small foot print) analytical equipment capable of operating at low power. It will also require reagents that, for the most part, show a tolerance to the environmental conditions in space, in particular radiation. Most of the current systems are fluid- and antibody-based, and the cartridges require refrigeration.

As with freezers, refrigerators for space vehicles (e.g. Merlin) provide ~20 l of storage in ~2 ft^3^ of volume at a maximum cold point of −20 °C (NASA Fact Sheet). Both of these requirements are unsuitable for exploration missions for the reasons outlined above. To combat the inherent fragility of antibodies in hostile conditions, new, more adaptable molecules are being used with considerable success.

We and our colleagues are pursuing X-Aptamer technology for the purpose of assessing a targeted proteome and metabolome on missions to the Moon and Mars. This will afford the ability to make real-time assessment of astronaut health during missions BLEO. X-Aptamers are small DNA fragments (oligonucleotides) resistant to denaturation. Like antibodies, they are made to specific protein epitopes, thus allowing a direct rather than indirect measure of the biomarker. Real-time evaluation affords protection of volatile analytes that might otherwise be degraded. X-Aptamers are coupled with a variety of chromophores, and have proven to be more tolerant to hostile environmental conditions and also to have sensitivity comparable to or greater than antibodies (Durland et al. 1991; He et al. 2012; Hecht et al. 2010).

With these attributes, aptamer reagents may be stored for years, rather than weeks, without refrigeration and yet assure reliability when needed. X-Aptamers can be designed to detect specific proteins as standard antibodies traditionally do. These can be measured during long-duration missions and results transmitted electronically to Earth for medical evaluation. Similar technologies will have to be developed with careful attention to parameters of instrument volume, reagent volume, reagent stability to radiation and temperature, and reliability in microgravity.

In general, the Omics technologies available today are fully ready to support development of personalized medicine in human space flight. Understanding the real-time molecular dynamics in long-duration missions will, however, require significant advances in the field, with regard to the space flight hardware. There is little doubt that these advances would also confer considerable value to the evolution of Earth-based technology and medicine.

### Systematic application of personalized medicine

The application of personalized medicine in human space flight seems inevitable given the vast pool of knowledge emerging from the field of systems biology and our growing understanding of individual susceptibility. Working from this assumption, it is imperative that a road map be developed that accounts for the present state of knowledge, as well as for the evolution of the field going forward. We suggest that a road map be developed with the goal of advancing Omics-based assessment and individualized countermeasures, as the foundation of medicine of the twenty first century for human space flight. This medicine would be personalized, preventive, predictive, and participatory.

The new paradigm will necessarily involve Earth-based mission-preparatory countermeasures, in-flight space-based countermeasures, habitation space-based countermeasures, and recovery-based countermeasures on return to Earth.

This would, at minimum, include: (1) establish the criteria for “best evidence” that can be used to develop individualized countermeasures today; (2) establish the criteria for best evidence that prioritizes research, clinical assessment, and individualized countermeasures to be developed in the near term; (3) establish a deliberate discovery path that seeks to develop sophisticated and more complex models for long-term deployment of personalized medicine as the standard in human space flight.

In summary, this review is not intended to proffer any policy statement or position, but rather to explore a series of core concepts from which personalized approaches to enhance astronaut performance, endurance, and safety can be developed. This new approach will require both scientific and technology advances, coupled with novel implementation strategies compelled by the rigors of extended duration missions and habitation in the final frontier. These methods applied to the complexity of space flight are also expected to become valuable tools, as we advance the personalized medicine paradigm in Earth-based medicine.
